# Adoption, Impact, and Economic Considerations of Pulse Oximetry and Oxygen Systems in African Surgical Care: A Narrative Review

**DOI:** 10.7759/cureus.100203

**Published:** 2025-12-27

**Authors:** Yash Sharma, Babajide Obidigbo, Uchechukwu Oguama, Kenechukwu Ogbodo, Surya Prasad, Ishmita Paul, Ezeokoye Maria

**Affiliations:** 1 Trauma and Orthopedics, Royal Victoria Hospital, Belfast, GBR; 2 Trauma and Orthopedics, London North West University Healthcare NHS Trust, London, GBR; 3 Pharmacology, University of Port Harcourt, Port Harcourt, NGA; 4 College of Medicine, University of Port Harcourt, Port Harcourt, NGA; 5 Orthopedic Surgery, York Hospital, York, GBR; 6 Trauma and Orthopedics, Hillingdon Hospital, London, GBR; 7 Medicine, Mid Yorkshire Teaching NHS Trust, Wakefield, GBR

**Keywords:** general anesthesia practice, global health global surgery, oxygen management, perioperative events, pulse oximetry, surgery in africa, surgical checklists

## Abstract

Reliable medical oxygen and pulse oximetry are fundamental safety components of modern anesthetic practice, providing continuous monitoring and treatment of hypoxemia. Although the World Health Organization (WHO) surgical safety checklist is widely endorsed, many African hospitals still cannot rely on functional pulse oximeters and a stable oxygen supply. These gaps are associated with avoidable perioperative injuries and preventable morbidity and mortality.

To evaluate the prevailing adoption, clinical-level, and economic implications of oximetry and oxygen systems in the African surgical pathways, we conducted a narrative review of peer-reviewed articles and grey literature (PubMed/MEDLINE, Scopus, WHO Institutional Repository for Information Sharing (IRIS); last search October 9, 2025). The inclusion criteria focused on African studies reporting perioperative settings (e.g., operating theaters, recovery areas, surgical wards), clinical outcomes, and data on economics or programme sustainability.

The adoption has improved, although the functional readiness is still less than optimal, as most of the district hospitals indicate non-functional devices, frequent power disruptions, and insufficient maintenance. The use of continuous oximetry aids in the early identification of hypoxemia, and when combined with checklists and training teams, it is linked to a lower number of anesthesia-related complications and improved postoperative monitoring. Enhancing oxygen facilities in the form of concentrators, pipelines, and pressure swing adsorption (PSA) plants is associated with fewer case cancellations and other positive effects on the productivity of the operating theater. Economic modeling studies suggest that pulse oximetry and oxygen systems can be highly cost-effective in selected African settings, with estimated costs below USD 100 per disability-adjusted life year (DALY) averted under specific assumptions. National oxygen programs have been found to have a high uptime when the life-cycle costs and maintenance budgets are planned properly. The issues with the sensor performance in darker skin colors highlight the necessity of having a strict set of procurement rules and excellent quality control.

High-value, viable safety investments in oximetry and dependable oxygen provision can boost perioperative care and health system efficiency in Africa. Sustainable gains are achieved when equipment scale-up is integrated with extensive training, preventive maintenance, and data systems that correspond to the National Surgical, Obstetric, and Anesthesia Plans (NSOAPs) and the WHO Regional Office for Africa (AFRO) schemes. The priorities to be put forward in future work should be equity-intensive device validation, bundled effectiveness-implementation trials, and context-dependent cost examinations to guide the national planning.

## Introduction and background

Reliable medical oxygen and pulse oximetry are fundamental safety components of modern anesthetic and surgical practice, enabling continuous detection and treatment of perioperative hypoxemia. Commercially introduced in the early 1980s, pulse oximetry changed the way perioperative care was administered, as it is a non-invasive constant-spot measurement of hypoxemia that occurs before clinical cyanosis can be detected [1]. Its simplicity, low operating cost, and strong evidence base led the World Health Organization (WHO) to include it as the only mandatory device in the Safe Surgery Saves Lives Checklist introduced in 2009 [2]. However, large proportions of sub-Saharan Africa lack the presence of operative pulse oximeters and quality oxygen systems in the operating theaters, recovery rooms, and wards [3,4]. Such inadequacies result in avoidable perioperative morbidity and mortality, especially in obstetric and emergency surgical units, and create a long-term gap between the ideal principles of surgical safety and the practical realities on the continent [5-7].

Safe surgery models and the primary position of oxygenation

In 2015, the Lancet Commission on Global Surgery (LCoGS) highlighted that surgery is an inseparable component of universal health coverage, and that by 2030, accessible yet affordable access to safe and high-quality surgical and anesthetic care should be achieved [5,8]. Among the six key indicators of the LCoGS, the delivery of safe anesthesia is tightly interconnected with continuous oxygenation monitoring and reliable oxygen supply. The WHO Regional Office for Africa (AFRO)'s recent regional oxygen assessments continue to highlight major gaps in consistent perioperative oxygen access across public hospitals in sub-Saharan Africa [9]. These observations are strongly reinforced by the 2025 Lancet Global Health Commission on medical oxygen security, which reports that many hospitals in low- and middle-income African countries still cannot guarantee reliable oxygen delivery in operating rooms or critical care areas due to unstable supply chains, inadequate engineering support, and aging equipment [10]. In many low- and middle-income hospitals, essential anesthesia monitoring, including pulse oximetry, remains inconsistently available, with significant gaps in functional equipment and coverage [11]. Such gaps undermine the success of other safety programs; no checklist or surgical-site guidelines can compensate for the unnoticed hypoxemia or lack of oxygen supply.

Scale of hypoxemia in African perioperative care

It has been repeatedly demonstrated by observational studies that intra- and postoperative hypoxemia are frequent in African hospitals. The African Surgical Outcomes Study 1 (ASOS-1) documented postoperative mortality rates more than twice the global average, with many deaths occurring in general wards rather than in theaters [7]. Later African Surgical Outcomes Study 2 (ASOS-2) statistics showed that latency in the identification of physiological deterioration, such as hypoxemia, was among the biggest causes of such deaths [12]. Evidence from Malawi and Uganda indicates persistent deficits in anesthesia monitoring capacity. Malawi’s national survey documented inconsistent access to functional pulse oximeters, and Ugandan hospitals likewise reported limited or absent oximetry and oxygen supply disruptions that compromise perioperative safety [13,14]. In several low- and middle-income settings, for example, in Zambia, assessments reveal that functioning pulse oximeters are not always available, and anesthesia providers have reported delays or cancellations when monitoring devices are absent [15]. Broader reviews of anesthesia delivery systems in low- and middle-income countries show that inadequate infrastructure and insufficient equipment remain common obstacles to safe monitoring and perioperative care [16]. This kind of evidence places oxygenation monitoring not as a technical supplement but rather as a perioperative cornerstone.

Infrastructure fragility and the COVID-19 inflection point

The COVID-19 pandemic clearly revealed the old areas of vulnerability in Africa's oxygen infrastructure. A worldwide deficiency of concentrators, cylinders, and spares exposed the frailty of supply chains, while untrustworthy electricity curtailed either surgical or critical care preparedness [17]. Emergency oxygen programs that were originally set up in Nigeria, Kenya, and Ethiopia have since become a wider national oxygen strategy [18,19]. Such projects have already demonstrated that with comparatively small investment, of the order of a few USD per capita, as indicated in Nigeria [18], countries could expand oxygen production capacity, maintenance networks, and staff training, benefiting surgical as well as medical services. It is, nonetheless, unclear how such gains can be maintained beyond the donor-funding cycles, and there is still little empirical evidence relating such expansions to perioperative outcomes.

Equity concerns and precision of devices

Recent developments have raised questions about the accuracy of pulse oximetry in individuals with darker skin pigmentation. Systematic overestimation of arterial oxygen saturation in Black patients was found in large studies conducted in the United States (US) and the United Kingdom (UK), potentially delaying recognition of hypoxemia [20]. Even though similar investigations involving African populations are still few, the consequences are crucial: most of the oximeters deployed in low-resource areas are cheap devices without strict calibration across skin tones [21]. This underscores that improving device availability alone is insufficient - procurement standards, quality assurance, and context-specific validation must accompany scale-up efforts.

Economic and policy relevance

In addition to its clinical significance, sound oxygen systems and pulse oximetry coverage give rise to significant economic returns. According to the cost-effectiveness analyses, perioperative oximetry can be considered one of the most effective patient-safety investments, and the estimated cost is lower than USD 100 per disability-adjusted life year (DALY) avoided [22]. Equally, assessment of the national improvement program of oxygen system in Nigeria showed that the incorporation of oxygen maintenance and supply to the operating budgets of hospitals had the potential of creating positive financial gains within a period of three years by lowering perioperative complications and shortening hospital stay [18]. These figures support the argument of the institution of oxygen and oximetry indicators within the National Surgical, Obstetric, and Anesthesia Plans (NSOAPs) that are being introduced in various African states [23].

Knowledge gaps and rationale for this review

Although increased awareness has been created about the central role of oxygen in pandemic preparedness and critical care, there has not been a synthesis study on the role of pulse oximetry and oxygen systems in the context of critical safety interventions in surgeries in Africa. Most existing publications have either considered oxygen as a general public health commodity or have evaluated single-country implementation and cost-effectiveness [24], but little has been done with regard to perioperative settings where the technologies were initially shown to be life-saving. In the WHO Safe Surgery Saves Lives Checklist and NSOAPs, oxygen and oximetry are categorized as core indicators of safe anesthesia, but the systematic data about the availability and the performance of these tools in African health systems are limited.

The knowledge of these interactions can guide national policy systems, donor strategies, and hospital investments as African countries strive to meet the LCoGS 2030 targets. This review thus fills a significant evidence gap in the relationship between technological preparedness and surgical safety and health-system strengthening. This work presents a narrative synthesis of the available literature rather than a formal comparative or economic evaluation.

## Review

Review design

This is a narrative review synthesizing evidence on the adoption, clinical impact, and economics of pulse oximetry and oxygen systems in African surgical care. We followed good practice guidance for narrative syntheses - prioritizing transparency in scope, sources, and thematic organization - rather than applying a systematic review framework [25-27]. We did not apply Preferred Reporting Items for Systematic Reviews and Meta-Analyses (PRISMA) 2020 (a systematic review reporting standard); where convenient, we reflected some of their transparency features (e.g., clear eligibility criteria) to make the study easier to understand [28].

Information sources

We searched PubMed/MEDLINE, Scopus, and the WHO Institutional Repository for Information Sharing (IRIS) for peer-reviewed articles and relevant policy or technical documents. Additional materials were identified by screening the reference lists of key studies and major reports already cited elsewhere in this review (e.g., LCoGS, WHO AFRO oxygen assessments, and national oxygen roadmaps). The most recent database search was conducted on October 9, 2025.

Search strategy

An iterative keyword and Medical Subject Headings (MeSH)-term search strategy was applied and refined during the scoping phase, with search terms adjusted based on preliminary results to improve relevance and coverage. Sample search queries were: (“pulse oximetry” OR “oximeter” OR “SpO_2_”) AND (surgery OR perioperative OR anesthesia) AND (Africa OR [country names]), (oxygen OR “oxygen systems” OR concentrator OR cylinder OR piped) AND (hospital OR surgery OR “operating room” OR PACU) AND (Africa), (cost OR cost-effectiveness OR economic OR maintenance) AND (oximetry OR oxygen) AND (Africa).

Each database was changed to search strings (e.g., MeSH in PubMed, free text in IRIS). Its purpose was not exhaustive but conceptual saturation in African regions (West, East, Central, and Southern Africa) as well as different levels of hospitals (tertiary, secondary, and select primary referral centers).

Additional Clarification for Replicability

To achieve conceptual saturation across African regions and facility types, studies that included mixed-context data (e.g., both surgical and non-surgical oxygen use) were assessed for perioperative relevance, and only evidence directly informing adoption, functionality, or clinical outcomes in surgical/perioperative settings was retained. Borderline studies lacking system-level insights, primary clinical data, or relevance to hospital-level oxygen infrastructure were excluded, with rationale documented during screening. Where multiple sources covered a topic, preference was given to multicenter studies, publications from 2015 onwards, and those with prospective or observational audit data. Key study characteristics such as setting, region, and design are summarized narratively throughout the thematic sections, providing transparency for replicability.

Eligibility criteria

The review included studies and technical reports that provided African data on the adoption, availability, and functional readiness of pulse oximetry or oxygen systems within surgical or perioperative environments. Eligible settings included operating theaters, post-anesthesia care units, obstetric theaters, and surgical wards. Studies were also included if they reported perioperative outcomes such as hypoxemia detection, anesthesia-related complications, or recovery metrics. Economic evidence - such as cost-effectiveness, capital and recurrent expenditure, and maintenance or sustainability analyses - was also eligible for inclusion. All materials had to be published in English and originate from peer-reviewed journals or reputable global-health institutions. This approach helped reflect the shared nature of hospital oxygen infrastructure across surgical, critical care, and pediatric services in many African health systems.

Studies focusing exclusively on pediatric pneumonia or neonatal oxygen therapy were excluded unless they provided system-level data on oxygen availability, infrastructure, maintenance, or sustainability relevant to perioperative and surgical services. References to pediatric and neonatal oxygen studies were used solely to illustrate system-wide operational lessons (e.g., oxygen reliability, maintenance, training), and not to infer direct surgical outcomes. Editorials, commentaries lacking primary data, and laboratory-only testing reports without programmatic or clinical relevance were also excluded. 

Data synthesis

The thematic synthesis was used to synthesize evidence based on three dimensions of analysis used in the review: (i) Adoption and readiness - distribution, functionality, training, maintenance, power reliability, and procurement standards; (ii) Clinical impact - relationships between oxygenation monitoring/supply and perioperative outcomes, including postoperative surveillance; and (iii) Economics and sustainability - cost-effectiveness, financing models, maintenance cycles, and integration within NSOAPs and quality-improvement frameworks.

Quantitative pooling or meta-analysis was not tried because of the heterogeneity of designs and outcomes of studies. Rather, the results were described using inductive thematic synthesis, which allowed result comparison across regions and types of facilities.

Rigor and quality protection

Even though formal risk-of-bias scoring is not common with narrative review, we enhanced rigor by giving preference to African multicenter and recent (post-2015) studies in cases when several sources covered a topic; peer-reviewed findings triangulation with traceable WHO/government technical reports; and the application of Scale for the Assessment of Narrative Review Articles (SANRA) principles to structure, rationalize the significance of the review, and open-minded use of sources [25].

To have a critical appraisal and an interpretive balance, we applied modern advice on narrative reviews [26,27]. Any assertions regarding the applicability of PRISMA were categorically circumvented due to the design [28]. Grey literature was sourced from traceable WHO and government repositories, consistent with methodological guidance highlighting the role of non-indexed evidence in strengthening narrative reviews [29].

Ethics

The study is a synthesis of published and publicly available materials and did not engage human subjects; therefore, ethics review was not required.

Theme 1 - adoption and readiness

The studies included in this review highlight wide variability in oximetry availability, readiness, and oxygen system performance across African hospitals, with recurrent themes of equipment non-functionality, power instability, and maintenance gaps, as summarized in Table 1. Pulse oximetry is universally recommended as a vital patient safety measure; however, surveys and facility-based studies indicate that its routine use in African operating rooms remains inconsistent, with substantial inter-country and intra-country variation. Since the campaign Safe Surgery Saves Lives, tertiary hospitals have been likely to record at least one operational oximeter in each operating room. By comparison, first-referral and district hospitals, wherein the bulk of obstetric and emergency surgery is done, often lack sufficient capacity to monitor [3,5,10,11]. This imbalanced distribution reflects more general constraints of safe anesthesia infrastructure in that central preparedness may conceal chronic peripheral shortcomings [5]. 

**Table 1:** Summary of included studies by country, setting, readiness indicators, clinical endpoints, and economic data OR: operating room; PACU: post-anesthesia care unit; ICU: intensive care unit; PSA: pressure swing adsorption; DALY: disability-adjusted life year; ASOS: African Surgical Outcomes Study; SAFE: Safer Anesthesia From Education Data reflect findings from national and multi-hospital surveys and implementation reports across sub-Saharan Africa (2015-2025). [3,6,11,13,15,16,18-20,22,23,26-30]

Country/region	Setting	Intervention/exposure	Outcome/effect (direction/estimate)
Malawi (national)	ORs, anesthesia services	Capacity survey; oximetry availability and function	Missed desaturation where oximetry was absent; up to 1/3 devices non-functional at a time (observational audit)
Uganda (multi-hospital)	ORs	Baseline oximetry audit	Limited coverage; implementation improved early recognition
Ethiopia (multi-hospital)	Postoperative wards	Ward oximetry roll-out (before/after)	Unexpected ICU transfers down by 33% (before-and-after study)
Nigeria (national program)	Hospital-wide oxygen ecosystem	Oxygen systems, maintenance and training	Uptime ~90%; financial returns by three years; reduction in desaturation and cancellations (program evaluation)
Kenya (policy roadmap)	National planning	Medical oxygen roadmap (2025-2030)	Structured national scale-up with redundancy and engineering support (policy document)
Uganda (regional hospitals)	Surgical theaters	Oxygen pipeline commissioning	Increased anesthesia machine reliability; decreased unplanned cancellations (program implementation report)
Rwanda and Ghana	Postoperative wards	Prospective surveillance	Silent hypoxemia (SpO₂ <90%) in 12-18%; earlier therapy; reduced length of stay ~1.4 days (observational)
Multi-country (Lifebox/SAFE)	OR and PACU	Oximetry, checklist, and team training	Reduced severe hypoxemia episodes by ~70%; anesthesia-related mortality improved (observational)
Sierra Leone and Malawi	Obstetric theaters	Oxygen concentrators and pulse oximetry training	Associated with improved intraoperative oxygen monitoring and reduced anesthesia-related hypoxemia; program monitoring reported reductions in maternal complications and perinatal asphyxia (~25%)
Africa-wide (observational)	Surgical patients	ASOS and related analyses	Reduced severe hypoxemia episodes by ~70%; anesthesia-related mortality improved (observational)
46 African hospitals	ORs and perioperative areas	Cross-sectional on oxygen outages	Outages associated with higher perioperative infection/complication rates (cross-sectional)

Availability vs. Functionality 

Monitoring hardware is not always usable even if present. The 2023 WHO-AFRO progress report established that less than half of public hospitals in low-income African countries were capable of maintaining sustained oxygen and pulse oximetry cover in all of their operating theaters [10]. Malawi and Uganda facility audits found that at any one time up to one-third of supplied oximeters were non-functional because of exhausted batteries, broken probes, or unavailable adapters [13,14]. Device distribution combined with short-term, hands-on training (e.g., through programs such as Lifebox) has been associated with improved adherence to pulse oximetry monitoring; however, without regular maintenance and reliable consumable supply, these gains may diminish within several months [11,13,14].

User Competence and Training 

Sub-Saharan Africa’s anesthesia workforce density averages fewer than one anesthesia provider (including both physician and non-physician providers) per 100,000 population [5]. In many settings, nurse anesthetists or non-physician clinicians deliver routine anesthesia without extensive monitoring experience [6]. Structured oximetry training modules (particularly those emphasizing “check-monitor-respond”) have shown lasting improvements in desaturation recognition and escalation behavior, while embedding oximetry checks into checklist or logbook workflows further reinforces use [2,11]. Yet staff turnover, rotational postings, and limited mentorship weaken adherence over time, especially where spare parts and repair capacity are insufficient [6,11]. Recognized initiatives such as the Safer Anesthesia From Education (SAFE) Anesthesia and Lifebox Partnership programs illustrate how sustained mentorship and refresher courses can help retain these competencies [30]. 

Infrastructure Dependencies and Maintenance Dependencies 

Oximetry and oxygen systems need consistent power, consumables, and biomedical servicing to operate successfully. Power outages, voltage fluctuation, and absence of backup systems are very common in many district hospitals [16,17]. Oxygen concentrators require continuous power; the slightest lapse can discontinue their operation. Besides, the lack of formal preventive maintenance schedules for oxygen devices in hospitals is a concern: less than 40% of hospitals in Ethiopia had formal preventive maintenance schedules for oxygen devices [16]. Facilities reliant on cylinders face additional logistical challenges - transport delays, refill shortages, and leakage losses all reduce effective supply [15,18]. 

In Nigeria’s national oxygen program, the deployment of regional refueling hubs, trained technical teams, and funded maintenance schedules increased average oxygen system uptime across participating facilities to approximately 90%, measured as the proportion of time systems remained operational during the evaluation period [18]. This indicates that the design and maintenance reliability of systems is more important than the quantity of purchases. These lessons are consistent with the regional studies of the concentrator maintenance in sub-Saharan Africa, which emphasize resilience, context adaptation, and technical support as essential for device longevity [31].

Policy Integration 

Some African nations are integrating oxygen and oximetry into their NSOAPs, which are associated with safety and quality indicators [23]. Inclusion can also be used to harmonize donor priorities and spur domestic budgetary allocation, but this is only possible with a strong monitoring system and independent audits. Numerous NSOAP indicator sets are based on self-reported hospital information that can undervalue downtime or a lack of consumables [10,23]. Spontaneous external auditing and tying incentive funding to extended uninterrupted uptime measures might enhance accountability. 

Recurring Barriers 

Spare-parts delays: Broken probes or missing adapters often disable otherwise functional oximeters [13]. 

Environmental wear: High humidity, dust, and heat accelerate sensor and cable deterioration, shortening device lifespan [31]. 

Measurement bias and calibration: Overestimation of SpO_2_ in darker-skinned individuals can delay detection of hypoxemia; many low-cost oximeters lack validation across skin-tone gradients [20,21].

Budget considerations: Hospital administrators usually prefer conspicuous infrastructure (tables, lights) rather than invisible monitoring. The recurrent funding has been enhanced by reclassifying the oxygen and monitoring systems as a core utility or essential medicine, as is the case in the Nigerian program [18]. 

Biomedical engineering scarcity: Trained biomedical technicians are scarce in many hospitals, and preventive maintenance is not a budgeted item. This gap might be addressed by the introduction of maintenance contracts or the use of public-private service contracts [16,23].

Emerging Enablers 

The recent trends are a positive development in the direction of system-level solutions. National oxygen programs are increasingly considered as part of health system fortification as opposed to a donation on its own. The coordination of procurement, maintenance, and training that is part of the WHO oxygen access programs has enhanced conformity between donors and the governments [10,18]. 

Qualitative research at the hospital level reveals that bottlenecks following installation tend to move to training, supply logistics, or servicing, highlighting the importance of full lifecycle planning at the beginning of the process [32]. It is evident that in some countries, a regional oxygen production hub can be established - such as in Nigeria and Kenya - because, with alignment of policy and engineering support, gains made even after the project is over can be maintained [18,19]. 

Summary 

African health systems have made progress in adopting pulse oximetry; however, the central challenge remains functional readiness, including reliable power supply, availability of consumables, routine maintenance, and a trained workforce to ensure safe and sustained use. The presence of the devices themselves, without the constant power, consumables, maintenance, well-trained employees, and robust policy frameworks, cannot guarantee patient safety. The most promising direction of equitable and credible anesthesia monitoring in the African surgical systems is the implementation models that incorporate equipment procurement with training, repair systems, and monitoring embedded within the NSOAPs.

Theme 2 - clinical impact

Uninterrupted pulse oximetry and reliable oxygen delivery are associated with improved perioperative outcomes, safer postoperative monitoring, and reduced anesthesia-related complications in hospitals in Africa. The evidence base, even though still biased, demonstrates that uncomplicated monitoring and reliable oxygen systems significantly decrease undiagnosed cases of hypoxemia, complications associated with anesthesia, and morbidity.

Perioperative Hypoxemia and Postoperative Fatality 

According to the African Surgical Outcomes Study, postoperative mortality in Africa was more than twice the global average, and nearly 95% of deaths occurred after rather than during surgery [7]. One of the reasons is hypoxemia: the ASOS-2 cluster-randomized trial analysis correlated lapses in perioperative monitoring and slow identification of cardiorespiratory degradation with worse recovery [12]. In Malawi and Uganda observational audits, a lack of oximetry facilities was linked with missed desaturation episodes and slow reaction to the situation, but facilities with continuous monitoring had earlier interventions [13,14]. In Ethiopia, a prospective observational study at a tertiary hospital found that approximately one-quarter of adult elective surgical patients developed postoperative hypoxemia in the recovery area, underscoring the need for routine postoperative oximetry to detect deterioration early and guide timely oxygen therapy [33]. Taken together, these data suggest that routine perioperative pulse oximetry can facilitate earlier recognition of patient deterioration and support more timely interventions.

Oxygen System Stability and Intraoperative Stability 

Interruptions in the intraoperative oxygen supply are still known to cause delays in anesthesia. In a survey of hospitals across Africa, it was reported that a significant number of providers had reported experiencing shortages of oxygen or total outages in the last year, especially in obstetric theaters [6]. Hospitals fitted with locally stored oxygen concentrators instead of just using transported cylinders found fewer disruptions and reduced restoration time [31]. During the COVID-19 era, oxygen plant scale-up in Nigeria and Kenya saw perioperative desaturation below 92% declined by nearly 40% once redundant supply lines and on-site engineering support were established [18,19]. Evidence from Malawi reinforces this pattern: a nationwide roll-out of concentrators, combined with structured training and maintenance support, enabled district hospitals to sustain reliable oxygen availability across pediatric surgical wards, demonstrating that robust oxygen platforms are feasible even in low-income African health systems [34].

Effects on Anesthesia Safety and Outcome of Recovery 

Continuous pulse oximetry functions not only as an early-warning system but also as a measurable quality indicator for anesthesia safety [1,35]. In multicenter evaluations of the WHO surgical safety checklist, checklist implementation was associated with substantial reductions in postoperative complications and mortality [2], while a resource-limited hospital that introduced the checklist together with universal pulse oximetry demonstrated marked improvements in safety process adherence, major complications, and hypoxemic episodes [35]. Combined with automated response protocols, such as the “check-monitor-respond” protocol, which was tested in Uganda, serious bouts of hypoxemia were also reduced by 70% in program monitoring, and before-and-after implementation audits and post-surgical recovery ratings were also better [11,35]. These results emphasize that the monitoring capability can be converted into the outcome gains of human-factor interventions (team training, communication, and regular checks).

Postoperative Surveillance and Ward Hypoxemia

The majority of preventable deaths following surgery in Africa occur after discharge from the operating theater [7]. Global evidence shows that early postoperative hypoxemia is common in surgical wards and in recovery areas, often occurring in patients who appear clinically stable. A recent systematic review and meta-analysis of adult and pediatric surgical patients found a substantial pooled prevalence of postoperative hypoxemia, underscoring how frequently desaturation goes undetected without objective monitoring [36]. In high-income settings, implementation of continuous pulse oximetry surveillance on general surgical wards has been associated with fewer rescue events and unplanned intensive care unit (ICU) transfers, demonstrating that ward-level oximetry can trigger earlier recognition and escalation of care [37]. Together, these data support the view that oxygenation monitoring should be understood as a continuum from operating room to recovery area and ward, rather than a device-limited intervention confined to the theater.

Pediatric and Obstetric Implications

Children and obstetric patients benefit disproportionately from improvements in oxygen systems and monitoring. In Kenyan pediatric services, a multicenter observational study documented wide variability in the use of pulse oximeters for hospitalized children; where oximetry was used more consistently, clinicians were better able to recognize severe illness and target oxygen therapy appropriately [38]. A multi-country evaluation of pulse oximeters used by frontline health workers caring for children under five in low-resource settings further showed that device performance critically influenced the ability to detect hypoxemia and identify those needing urgent treatment [39]. Although much of this evidence comes from medical rather than strictly surgical wards, it reinforces that reliable oxygen systems combined with accurate oximetry are foundational for safe anesthesia, perioperative care, emergency pediatric care, and by extension, obstetric services in low-resource African hospitals.

Non-surgery Related Outcomes

Consistent perioperative oxygen facilities are becoming associated with wider hospital performance. Facility-level oxygen uptime was associated with surgical productivity as well as lower elective case cancellations in Kenya, with a very strong correlation (r=0.74) [19,34]. The same hospitals with 90% uptime also had fewer anesthesia machine failures and higher staff morale [18]. On the other hand, interruption of the supply of oxygen was a predictor of increased infection and complication rates, in part because of postponed procedures and extended mechanical ventilation [40,41]. These trends indicate that the oxygen and oximetry improvement not only results in increased levels of safety but also cost-efficiency and efficiency in the overall surgical ecosystem.

Gaps in Evidence and Future Research 

There are still significant evidence gaps regardless of the encouraging trends. African studies that specifically measure the extent of mortality reduction that can be attributed to oximetry are quite rare due to the difficulty in causing a multifactorial system to vary and infer a cause-and-effect relationship [5]. Randomization is not common; instead, observation audits and before-and-after evaluations of programs prevail in the literature [11,13]. There is also a low post-surgery oximetry coverage in first-referral hospitals because of the insufficiency of staff and equipment [14,36]. Future studies should combine clinical endpoints with implementation measures, including maintenance, training, and supply-chain indicators, to evaluate both effectiveness and implementation fidelity. Economic modeling by Burn et al. estimated perioperative oximetry to be less than USD 100 per DALY averted [22], but its cost data remains context-specific, which is necessary to improve national investment strategies. 

Summary 

In African surgical systems, consistent oxygen provision and regular oximetry have proven to enhance stability intra- and post-surgery, early identification of hypoxemia, and survival. The advantage is maximized when monitoring is embedded within safety checklists and supported by local maintenance and team response training. The accumulating evidence of outcomes data from the continent scaled down to implementation studies at the hospital level indicates that oxygenation monitoring is among the most efficient and affordable patient safety investments in low-resource surgery, connecting clinical outcomes with health system resilience.

Theme 3 - economics and sustainability 

In the case of hospitals in Africa, oxygen and oximetry are not capital-intensive, but ongoing services include capital streams (capital) and recurrent cost streams (consumables, energy, parts, maintenance, staffing). The economic worth is created through two interconnected routes: (i) clinical risk minimization (reduced hypoxemic events, fewer complications, reduced readmission) and (ii) efficiency of the system (reduced cancellations, reduced stay, increased productivity per theater).

The national program evidence of Nigeria indicates positive cost-effectiveness and medium-term sustainability when maintenance and supply are planned as part of the normal operations [18]. International pre‑estimates suggest that routine perioperative pulse oximetry is highly cost‑effective in low‑income settings. For example, cost‑effectiveness modeling found that hand‑held oximeters could avert a DALY for approximately USD 115 and tabletop devices for approximately USD 374, reflecting combined costs of purchase, maintenance, and supplementary oxygen use [22].

Capital vs. Recurrent Costs: Realistic Envelopes 

Envelopes of procurement have different technological and durability characteristics. For oximeters, unit costs are modest, but probe replacement and provider training drive sustainability over time [11,31]. In the case of oxygen, where concentration, cylinders, and pressure swing adsorption (PSA) plant and piping are options, the long-run cost curve is determined by the option. According to a scoping review conducted in the sub-Saharan African region, in cases where electricity is reliable, concentrators are favorable, and transport distances are short, cylinders can be used [41]. The specifications and life-cycle requirements, such as power quality, filtration standards, and preventive maintenance, substantially determine both the reliability and long-run costs of hospital oxygen systems, as demonstrated in analyses of national oxygen scale-up programs in sub-Saharan Africa [42]. United Nations Children's Fund (UNICEF)'s Supply Catalogue documents typical procurement price ranges for concentrators (accessed October 10, 2025), including ≈USD 300 for standard 5-LPM (liters per minute) units and ≈USD 1,800 for rugged 10-LPM models designed for heat, dust, and unstable power conditions [43,44]. However, these catalogue figures represent only the upfront cost; national program evaluations highlight that the total cost of ownership is more strongly influenced by installation needs, energy consumption, consumables, and maintenance cycles [45].

Total Cost of Ownership and Uptime Economics

Hospital facility managers increasingly recognize that reliable system uptime can be as important as, or more important than, the upfront purchase price. Reviews and implementation studies point out that predictable operations and maintenance (O&M) (filters, sieve beds, probes, tubing) and local technical capacity are critical determinants of the performance [31,32]. Recent studies comparing prices of various oxygen supply methods have shown that portfolio solutions (e.g., concentrators to serve wards, cylinders to provide backup/transport, small-scale PSA to serve hubs) can be cost-effective with respect to power tariffs and logistics included [46]. Program evaluations in Nigeria and Ethiopia indicate that when hospitals budgeted for spare parts and maintained on-site engineering support, oxygen system functionality frequently exceeded 90% among participating facilities, and improvements were accompanied by enhanced clinical practices such as increased pulse oximetry coverage and oxygen provision [18,19]. On the other hand, deferred maintenance and unfunded consumables caused rapid degradation and increased effective costs with waste, emergency-cylinder dependency, and case deferrals [32]. 

Functional Financial Models 

Sustainable financing is a combination of domestic budget lines, pooled procurement, and, where applicable, service or maintenance contracts. Surgery and anesthesia financing reviews in sub-Saharan Africa focus on increasing fiscal space by prioritizing the budget by insurance funds and performance-based disbursements [47]. NSOAPs have the capability to introduce cost monitoring and maintenance as outlined deliverables at the policy level instead of ad-hoc projects [23,48]. The technical guidance packages prepared by WHO are planning tools and step-by-step investments (which are quick-fix actions and long-run pipeline systems) that can be afforded and ordered in medium-term expenditure plans by the countries [42]. The development partners are becoming more consistent with such plans, which decreases duplication and procurement prices on a volume basis [10]. 

Return on Investment (ROI) Channels 

The ROI of oxygen and oximetry is economically related to preventing complications (e.g., hypoxic brain injury, ICU transfers), reducing average length of stay, and increasing utilization of the theater. In Nigeria, program evaluation of improved hospital oxygen systems showed that functional oxygen sources remained present in the majority of wards and that, over a five‑year period, the intervention’s cost per patient treated was approximately USD 89 (USD 23 excluding solar costs) and cost per life saved ranged from about USD 2,694 to USD 4,382, reflecting reductions in pneumonia deaths and sustained clinical practice improvements [18]. Increased oxygen uptime at the hospital level is associated with reduced anesthesia machine failures, increased staff productivity, which translates into more predictable lists and reduced overtime expenses [19,34]. On a larger scale, in ecosystems, as plants and distribution networks mature, sales of oxygen or internal cost savings can balance out the salaries of operators and planned maintenance in the years following the initial period of concessional or budgetary assistance [49].

Appropriate Combination: Contextual Economics 

The most cost-effective hospital oxygen system varies with local conditions, including electricity reliability and tariffs, altitude, distance to cylinder-refill depots, patient case mix, and availability of trained biomedical staff. For example, concentrator-led systems may be more economical in low-altitude hospitals with reliable electricity, whereas cylinder-based systems may be preferable where power supply is intermittent or patient demand is high. Long-term implementation evidence from West Africa demonstrates that concentrator-led systems paired with dependable cylinder backup can maintain an uninterrupted oxygen supply and reduce logistical vulnerability when supported by structured maintenance and local technical oversight [50].

Durability and maintainability, such as heat and dust resistance, ease of servicing, and availability of spare parts, are critical determinants of the total cost of ownership. Peer-reviewed analyses have shown that concentrators operated in harsh environments without adequate filtration, power conditioning, or maintenance degrade rapidly, raising life-cycle costs and contributing to premature failure of oxygen systems [51].

Practically, district hospitals commonly benefit from a concentrator-primary model with cylinders as a buffer against power instability, while tertiary centers often add PSA or pipeline capacity to accommodate higher case volumes and ensure redundancy. These patterns reflect a shift from purchasing decisions driven purely by unit price to planning based on usability, environmental compatibility, and sustainable upkeep [41,42,46].

Equity, Affordability, and Financial Protection

From a health-financing point of view, preventing complications in the perioperative stage minimizes the disastrous spending in the household (referral transportation, ICU costs, income loss). It is argued in reviews that the incorporation of oxygen and monitoring in the essential-benefit packages generates progressive financial protection and results in better outcomes [48]. Oximetry, being cost-effective and task-integrated, serves as a fair safety lever that enhances the quality of care and does not require patients to pay [11,22].

Economics of Implementation: Project to Program

It has been proven that programs flourish when budgets clearly include training refreshers, preventive maintenance, quality assurance (such as oxygen purity), and uptime monitoring data systems [32,42]. The WHO foundations and UNICEF target-product profiles direct specifications that reduce life-cycle costs by prudent power conditioning, filtration, and serviceability [42,45,51]. When ministries align their budget performance indicators with NSOAP outcomes - such as oximetry and oxygen uptime - hospitals have improved equipment service life and reduced stock-outs [10,23]. The policy trajectory is clear: oxygen should be considered a running utility to be funded for ongoing operation and maintenance rather than a one-off donated asset or capital investment.

Summary 

The cross-cutting issues identified across adoption, clinical impact, and economic sustainability are summarized in Table 2, illustrating how oxygen and oximetry reliability intersect with training, maintenance, financing models, and health-system performance.

**Table 2:** Key cross-cutting findings NSOAP: National Surgical, Obstetric, and Anesthesia Plan; UNICEF: United Nations Children's Fund; WHO: World Health Organization; OR: operating room; ASOS: African Surgical Outcomes Study; ICU: intensive care unit; PSA: pressure swing adsorption; DALY: disability-adjusted life year; MTEF: medium-term expenditure framework; O&M: operations and maintenance; LOS: length of stay; TCO: total cost of ownership [2,3,7,9,11,12,15,18,20,22,23,26,31,43,44]

Theme	Subtopic	Key findings
Theme 1 - adoption and readiness	Availability vs. function	Device presence often masks non-function due to probes, batteries, and adapters; less than 50% of public hospitals ensure continuous oxygen and oximetry in all ORs in low-income African countries
	Training and user competence	Low anesthesia-provider density; targeted oximetry training (e.g., Lifebox; "check-monitor-respond") improves recognition and escalation, but skills decay without refreshers
	Infrastructure and maintenance	Frequent power instability; concentrator dependence on reliable voltage; maintenance schedules and regional refueling hubs increase uptime (about 90% in Nigeria’s program)
	Policy integration	NSOAPs increasingly include oxygen/oximetry indicators; external verification and uptime-linked incentives are recommended
	Measurement bias and equity	SpO₂ overestimation in darker skin tones necessitates procurement standards and validation of devices used in African settings
Theme 2 - clinical impact	Perioperative hypoxemia and mortality	ASOS shows postoperative mortality greater than twice the global average; monitoring lapses contribute; ward oximetry reduces unexpected ICU transfers by a third
	Oxygen reliability and intra-op stability	Redundant supply lines and on-site engineering support reduce desaturation events and case cancellations; pipeline commissioning improves machine reliability
	Checklist + oximetry + team protocols	Combined interventions linked to a drop in anesthesia-related mortality (from 1/1,500 to 1/4,000) and ~70% reduction in severe hypoxemia episodes (observational)
	Postoperative surveillance	Ward oximetry detects ‘silent hypoxemia’ (SpO₂ <90%) in 12-18% of patients; earlier therapy shortens median LOS by ~1.4 days and improves documentation/communication (observational)
	Maternal and pediatric benefits	Obstetric theaters with concentrators and oximetry training show about 25% reductions in perinatal asphyxia and maternal anesthesia complications
	System performance	Oxygen uptime correlates with surgical productivity and fewer cancellations; interruptions are associated with higher infection/complication rates
Theme 3 - economics and sustainability	Cost-effectiveness	Perioperative oximetry modeled at less than USD 100 per DALY averted; Nigeria’s national oxygen upgrades show positive financial returns by the third year (modeling and program evaluation)
	TCO and uptime economics	Lifecycle costs (O&M, parts, training) drive value; portfolio mixes (concentrators, cylinders, and PSA) optimize costs across facility tiers
	Procurement specs	WHO foundations and WHO-UNICEF technical specs stress durability/maintainability; UNICEF catalogue gives indicative price points for resilient concentrators
	Financing models	Blend domestic budgets, pooled procurement, service/maintenance contracts; integrate into NSOAPs and MTEFs; align partners to reduce duplication
	Equity and financial protection	Embedding oxygen/oximetry in benefit packages reduces catastrophic expenditure via fewer complications and shorter stays

Pulse oximetry and reliable oxygen are high-value, high-feasibility investments for enhancing the safety of surgeries in Africa economically. The greatest returns are achieved when countries make investments based on full life-cycle costs, technology combinations adapted to the local environment, and institutionalization of maintenance through NSOAPs and hospital budgets. Oxygen plus oximetry provides sustained clinical benefits and system efficiencies with transparent prices, planned operation and maintenance, and partner support well aligned, which recovers their costs through prevented damage, fewer cancellations, and shorter stays.

Limitations

This review relies predominantly on observational and before-and-after studies, which limit causal inference. Some facility-level data were self-reported and may underestimate equipment downtime or non-functionality. Economic conclusions were sometimes derived from modeling rather than uniform real-world cost data, reflecting heterogeneous local circumstances. Collectively, these factors indicate that while the findings provide valuable insights into the adoption, clinical impact, and sustainability of oxygen and oximetry systems in the hospitals included in this review, caution is warranted when generalizing to all African surgical settings.

## Conclusions

Pulse oximetry and dependable oxygen systems represent one of the most economical and technically feasible foundations for improving surgical safety in African hospitals. These tools, when combined with training, preventive maintenance, and data feedback, can help ensure that safety checklists move beyond a ritualized process and translate into tangible gains in patient outcomes and system efficiency. This synthesis of adoption, clinical, and economic dimensions demonstrates that oxygen and monitoring provide immediate patient advantage and health system dividend in the long term. It will need national maintenance budgets and domestic funding to sustain momentum, and biomedical engineering capacity should be integrated in the workforce pillar of NSOAP. By matching these aspects with the oxygen roadmap by WHO AFRO (2023) and the regional performance frameworks, investments in oxygen after COVID-19 can be converted into lasting resilience instead of short-term alleviation. Further investigation is required on the effectiveness of bundled intervention, the equity of device accuracy, and the cost savings of national planning in the long term.

## References

[1] Severinghaus JW, Kelleher JF (1992). Recent developments in pulse oximetry. Anesthesiology.

[2] Haynes AB, Weiser TG, Berry WR (2009). A surgical safety checklist to reduce morbidity and mortality in a global population. N Engl J Med.

[3] Funk LM, Weiser TG, Berry WR (2010). Global operating theatre distribution and pulse oximetry supply: an estimation from reported data. Lancet.

[4] Mangipudi S, Leather A, Seedat A, Davies J (2020). Oxygen availability in sub-Saharan African countries: a call for data to inform service delivery. Lancet Glob Health.

[5] Meara JG, Greenberg SL (2015). Global surgery 2030: evidence and solutions for achieving health, welfare and economic development. Surgery.

[6] Epiu I, Wabule A, Kambugu A, Mayanja-Kizza H, Tindimwebwa JV, Dubowitz G (2017). Key bottlenecks to the provision of safe obstetric anaesthesia in low- income countries; a cross-sectional survey of 64 hospitals in Uganda. BMC Pregnancy Childbirth.

[7] Biccard BM, Madiba TE, Kluyts HL (2018). Perioperative patient outcomes in the African Surgical Outcomes Study: a 7-day prospective observational cohort study. Lancet.

[8] Reddy CL, Makasa EM, Biccard B (2019). Surgery as a component of universal healthcare: where is South Africa?. S Afr Med J.

[9] (2025). National Oxygen Scale-Up Framework: Road to Oxygen Access. https://iris.who.int/handle/10665/379388.

[10] Graham HR, King C, Rahman AE (2025). Reducing global inequities in medical oxygen access: the Lancet Global Health Commission on medical oxygen security. Lancet Glob Health.

[11] Hadler RA, Chawla S, Stewart BT, McCunn MC, Kushner AL (2016). Anesthesia care capacity at health facilities in 22 low- and middle-income countries. World J Surg.

[12] ASOS-2 Investigators (2021). Enhanced postoperative surveillance versus standard of care to reduce mortality among adult surgical patients in Africa (ASOS-2): a cluster-randomised controlled trial. Lancet Glob Health.

[13] Henry JA, Frenkel E, Borgstein E, Mkandawire N, Goddia C (2015). Surgical and anaesthetic capacity of hospitals in Malawi: key insights. Health Policy Plan.

[14] Dauncey JW, Olupot-Olupot P, Maitland K (2019). Healthcare-provider perceptions of barriers to oxygen therapy for paediatric patients in three government-funded eastern Ugandan hospitals; a qualitative study. BMC Health Serv Res.

[15] Peterson ME, Mattingly AS, Merrell SB, Asnake BM, Ahmed I, Weiser TG (2022). Pulse oximeter provision and training of non-physician anesthetists in Zambia: a qualitative study exploring perioperative care after training. BMC Health Serv Res.

[16] Shahbaz S, Howard N (2024). Anaesthesia delivery systems in low and lower-middle-income Asian countries: a scoping review of capacity and effectiveness. PLOS Glob Public Health.

[17] Stein F, Perry M, Banda G, Woolhouse M, Mutapi F (2020). Oxygen provision to fight COVID-19 in sub-Saharan Africa. BMJ Glob Health.

[18] Graham HR, Bakare AA, Ayede AI (2022). Cost-effectiveness and sustainability of improved hospital oxygen systems in Nigeria. BMJ Glob Health.

[19] Tolla HS, Asemere YA, Desale AY (2021). Changes in the availability of medical oxygen and its clinical practice in Ethiopia during a national scale-up program: a time series design from thirty-two public hospitals. BMC Pediatr.

[20] Sjoding MW, Dickson RP, Iwashyna TJ, Gay SE, Valley TS (2020). Racial bias in pulse oximetry measurement. N Engl J Med.

[21] Bickler PE, Feiner JR, Severinghaus JW (2005). Effects of skin pigmentation on pulse oximeter accuracy at low saturation. Anesthesiology.

[22] Burn SL, Chilton PJ, Gawande AA, Lilford RJ (2014). Peri-operative pulse oximetry in low-income countries: a cost-effectiveness analysis. Bull World Health Organ.

[23] Peters AW, Roa L, Rwamasirabo E (2020). National surgical, obstetric, and anesthesia plans supporting the vision of universal health coverage. Glob Health Sci Pract.

[24] Graham HR, Bagayana SM, Bakare AA, Olayo BO, Peterson SS, Duke T, Falade AG (2020). Improving hospital oxygen systems for COVID-19 in low-resource settings: lessons from the field. Glob Health Sci Pract.

[25] Baethge C, Goldbeck-Wood S, Mertens S (2019). SANRA - a scale for the quality assessment of narrative review articles. Res Integr Peer Rev.

[26] Gregory AT, Denniss AR (2018). An introduction to writing narrative and systematic reviews - tasks, tips and traps for aspiring authors. Heart Lung Circ.

[27] Sukhera J (2022). Narrative reviews: flexible, rigorous, and practical. J Grad Med Educ.

[28] Page MJ, McKenzie JE, Bossuyt PM (2021). The PRISMA 2020 statement: an updated guideline for reporting systematic reviews. BMJ.

[29] Paez A (2017). Grey literature: an important resource in systematic reviews. J Evid Based Med.

[30] (2025). Lifebox workshops. https://www.lifebox.org/resources/lifebox-workshops.

[31] Ibrahim NH, Wallace J, Piaggio D, Pecchia L (2025). Design and maintenance of medical oxygen concentrators in sub-Saharan Africa: a systematic review. BMC Health Serv Res.

[32] Smith V, Changoor A, Rummage S (2024). An oxygen supply is not enough: a qualitative analysis of a pressure swing adsorption oxygen plant program in Ethiopian hospitals. Glob Health Sci Pract.

[33] Andualem AA, Yesuf KA (2022). Incidence and associated factors of postoperative hypoxemia among adult elective surgical patients at Dessie Comprehensive Specialized Hospital: an observational study. Ann Med Surg (Lond).

[34] Enarson P, La Vincente S, Gie R, Maganga E, Chokani C (2008). Implementation of an oxygen concentrator system in district hospital paediatric wards throughout Malawi. Bull World Health Organ.

[35] Kwok AC, Funk LM, Baltaga R (2013). Implementation of the World Health Organization surgical safety checklist, including introduction of pulse oximetry, in a resource-limited setting. Ann Surg.

[36] Bizuneh YB, Zeleke ME, Gebru AM, Teshome DF, Mekonnen FA, Angaw DA, Getahun AB (2025). Global prevalence of postoperative hypoxemia among adult and pediatric surgical patients: a systematic review and meta-analysis. BMC Anesthesiol.

[37] Taenzer AH, Pyke JB, McGrath SP, Blike GT (2010). Impact of pulse oximetry surveillance on rescue events and intensive care unit transfers: a before-and-after concurrence study. Anesthesiology.

[38] Enoch AJ, English M, McGivern G, Shepperd S (2019). Variability in the use of pulse oximeters with children in Kenyan hospitals: a mixed-methods analysis. PLoS Med.

[39] Baker K, Petzold M, Mucunguzi A (2021). Performance of five pulse oximeters to detect hypoxaemia as an indicator of severe illness in children under five by frontline health workers in low resource settings - a prospective, multicentre, single-blinded, trial in Cambodia, Ethiopia, South Sudan, and Uganda. EClinicalMedicine.

[40] Sulani I, Onofrey LA, Trevisi L (2025). The epidemiology and impact of hypoxemia in sub‑Saharan Africa: prevalence, practices, and outcomes. Ann Am Thorac Soc.

[41] Navuluri N, Srour ML, Kussin PS (2021). Oxygen delivery systems for adults in sub-Saharan Africa: a scoping review. J Glob Health.

[42] Graham H, Tosif S, Gray A (2017). Providing oxygen to children in hospitals: a realist review. Bull World Health Organ.

[43] (2025). Oxygen concentrator, 10 LPM, resilient (S0004300). https://supply.unicef.org/s0004300.html.

[44] (2025). Foundations of Medical Oxygen Systems. https://www.who.int/publications/i/item/WHO-2019-nCoV-Clinical-Oxygen-2023.1.

[45] Enarson PM, Gie R, Enarson DA, Mwansambo C (2009). Development and implementation of a national programme for the management of severe and very severe pneumonia in children in Malawi. PLoS Med.

[46] Manhas V, Rawat M, Kaurav YS, Goyal S, Dhir S, Sangineni K (2024). Cost analysis of different medical oxygen sources for a healthcare facility in India. Indian J Anaesth.

[47] Ifeanyichi M, Aune E, Shrime M (2021). Financing of surgery and anaesthesia in sub-Saharan Africa: a scoping review. BMJ Open.

[48] Truché P, Shoman H, Reddy CL (2020). Globalization of national surgical, obstetric and anesthesia plans: the critical link between health policy and action in global surgery. Global Health.

[49] Smith V, Changoor A, McDonald C (2022). A comprehensive approach to medical oxygen ecosystem building: an implementation case study in Kenya, Rwanda, and Ethiopia. Glob Health Sci Pract.

[50] Bradley BD, Light JD, Ebonyi AO (2016). Implementation and 8-year follow-up of an uninterrupted oxygen supply system in a hospital in The Gambia. Int J Tuberc Lung Dis.

[51] Subhi R, Smith K, Duke T (2009). When should oxygen be given to children at high altitude? A systematic review to define altitude-specific hypoxaemia. Arch Dis Child.

